# Supplementary material for: A conserved role for the ALS-linked splicing factor SFPQ in repression of pathogenic cryptic last exons
Source: Nat Commun. 2021 Mar 26;12:1918. doi: 10.1038/s41467-021-22098-z (PMC7997972; doi:10.1038/s41467-021-22098-z)
Supplement: Supplementary file 1 — Supplementary Figures [file 41467_2021_22098_MOESM1_ESM.pdf]

Supplementary Information:

A conserved role for the ALS-linked splicing factor SFPQ in repression of pathogenic cryptic last exons

by Gordon et al

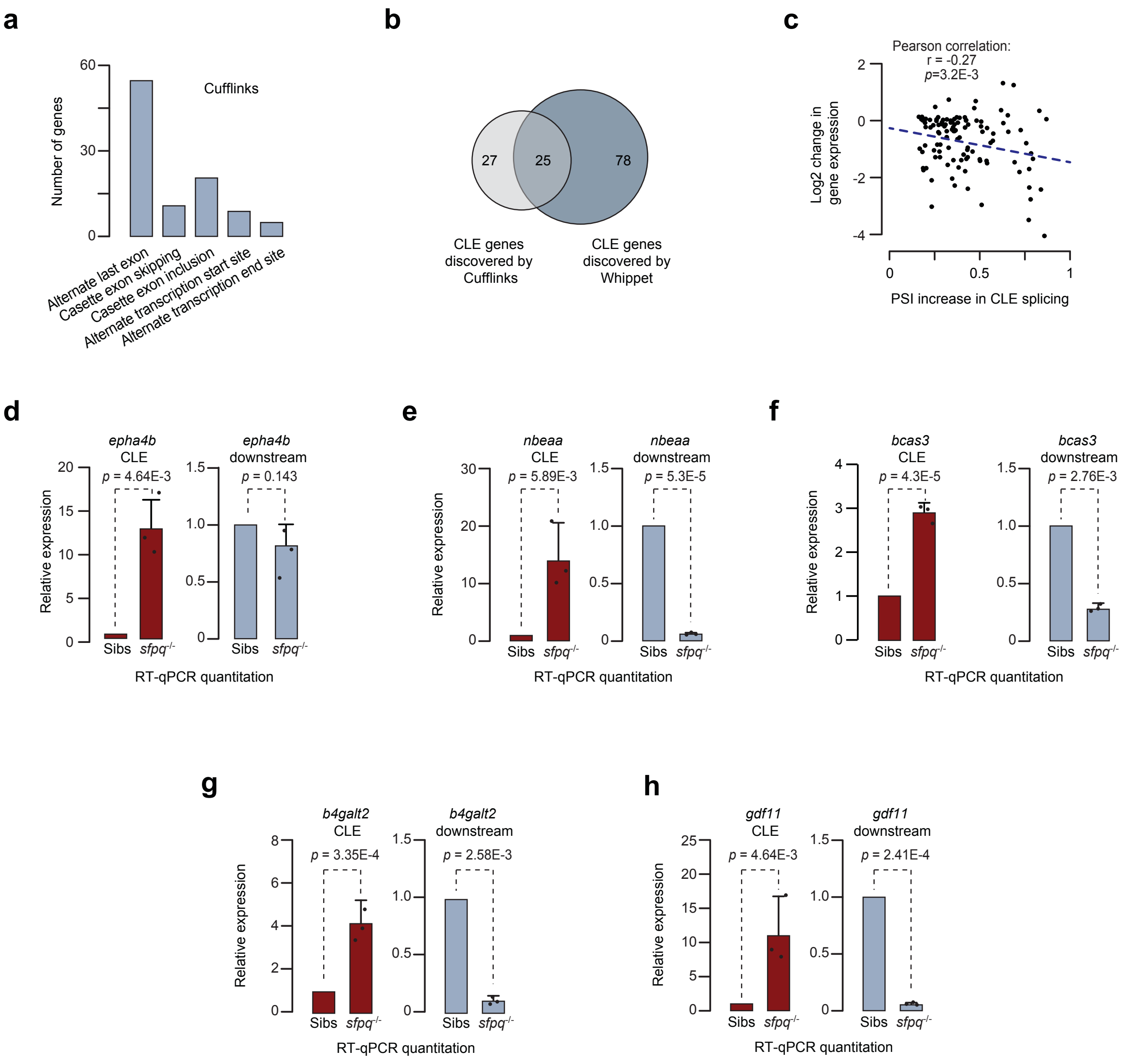

Supplementary Figure 1: SFPQ regulates the formation of cryptic last exons (CLEs)

a, Bar plot of splicing switches in significantly regulated genes from Cufflinks.

b, Total CLE-containing genes found using Cufflinks and Whippet pipelines

c, Correlation plot between the increase in CLE splicing versus change in expression of the same gene. Two-tailed Pearson's correlation test was performed.

d-h, RT-qPCR quantitation on the relative expressions of CLE-containing isoforms and CLE-lacking isoforms ("downstream") normalized to β-actin. Expression levels in "Sibs" were set to 1 and mean values from three replicates are shown (± SD). Two-tailed unpaired t-test was performed.

Source data are provided as a Source Data file.

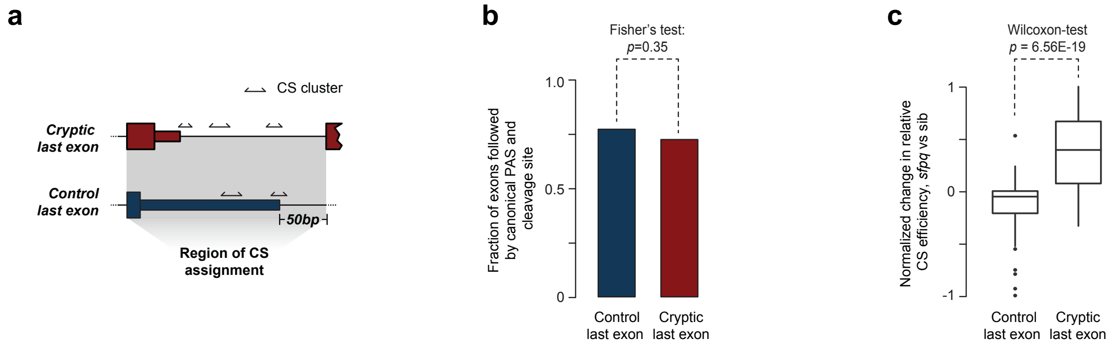

Supplementary Figure 2: CLE-terminated transcripts are cleaved and polyadenylated at the 3' end

a, Illustration on the method of assigning cleavage site (CS) clusters, analysed from 3’ mRNA-seq, to cryptic last exons or control last exons

b, Bar plot showing the proportion of cryptic or control last exons that are terminated with a CS cluster and a canonical polyadenylation signal (PAS). Two-sided Fisher’s-exact test was performed.

c, Box plot showing distribution in the relative difference in 3’ termination (sfpq vs sib) of cryptic (n = 94) and control last exons (n = 84). The box bounds represent the first and third quartiles and the black lines at the middle of the boxes show the medians. Top and/or bottom whiskers represent 1.5x of the range between the third and the first quartiles (interquartile range). Circles represent outliers. Two-sided Wilcoxon rank sum test was performed.

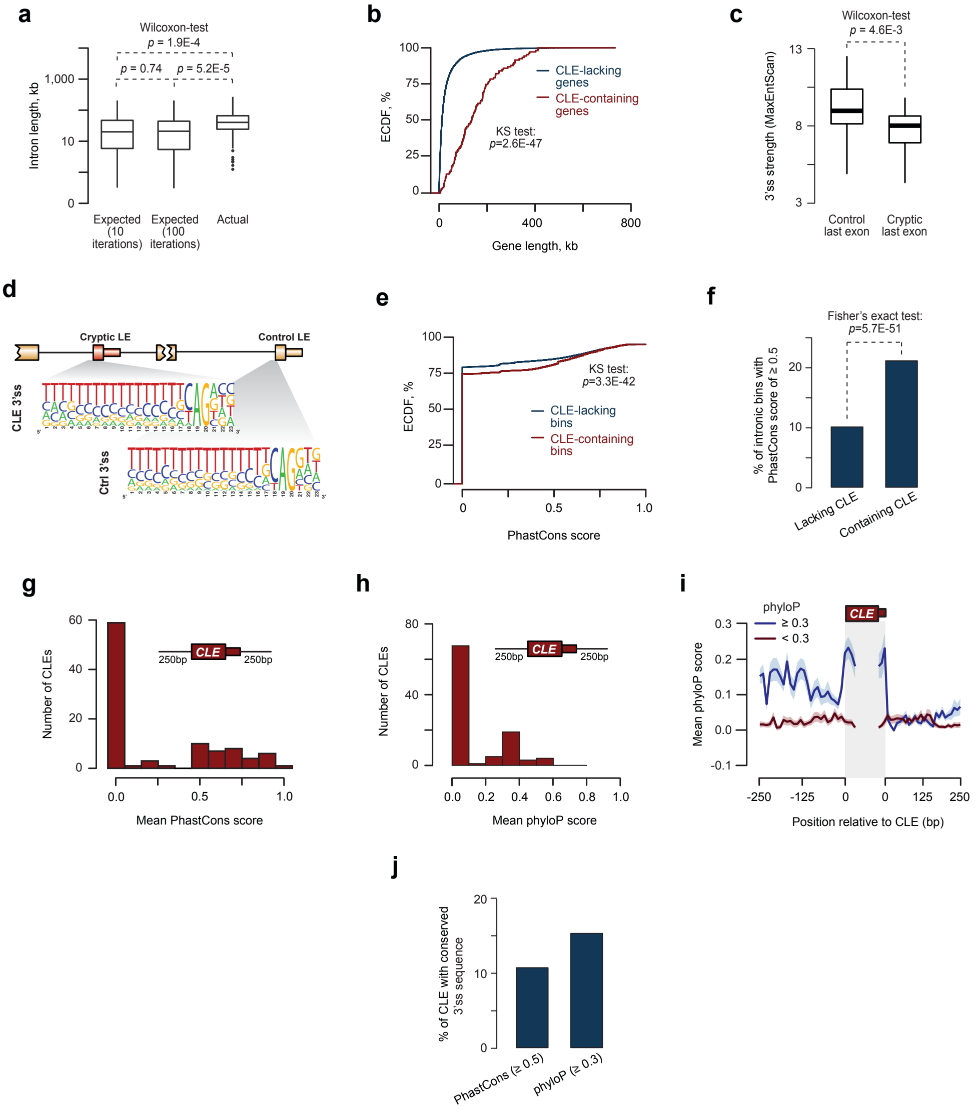

(Supplementary Figure 3. See next page for legend)

Supplementary Figure 3: Properties of CLE

a, Distribution of intron lengths from CLE-containing introns (n = 109) or from introns sampled from weighted random sampling method (n = 109). The box bounds represent the first and third quartiles and the black lines at the middle of the boxes show the medians. Top and/or bottom whiskers represent 1.5x of the range between the third and the first quartiles (interquartile range). Circles represent outliers. Two-sided Wilcoxon rank sum test was performed.

b, ECDF plot of gene lengths.

c, Boxplot showing the distribution of acceptor splice site strengths from region upstream of cryptic (n = 109) or constitutive (control) last exons. The box bounds represent the first and third quartiles and the black lines at the middle of the boxes show the medians. Top and/or bottom whiskers represent 1.5x of the range between the third and the first quartiles (interquartile range). Two-sided Wilcoxon rank sum test was performed.

d, Consensus 3' splice site sequence from cryptic (CLE) or control last exons are shown as WebLogo.

e, ECDF plot of mean PhastCons scores of bins (1 kb sliding window) along CLE-containing introns. Two-tailed Kolmogorov–Smirnov test was performed.

f, Bar plot of the fraction of intronic bins with ≥ 0.5 PhastCons score. Two-sided Fisher's-exact test was performed.

g, Histogram of mean PhastCons scores of CLE and 250 bp neighbouring its introns.

h, Histogram of mean phyloP scores of CLE and 250 bp neighbouring its introns.

i, Metaplot of mean phyloP scores of region surrounding CLEs. CLEs have been grouped based on its overall score in (h).

j, Bar plot showing the proportion of CLEs with conserved 3' splice acceptor sequence based on PhastCons and phyloP scoring models. Sequence exceeding a cutoff of 0.5 (PhastCons) or 0.3 (phyloP) are categorised as conserved.

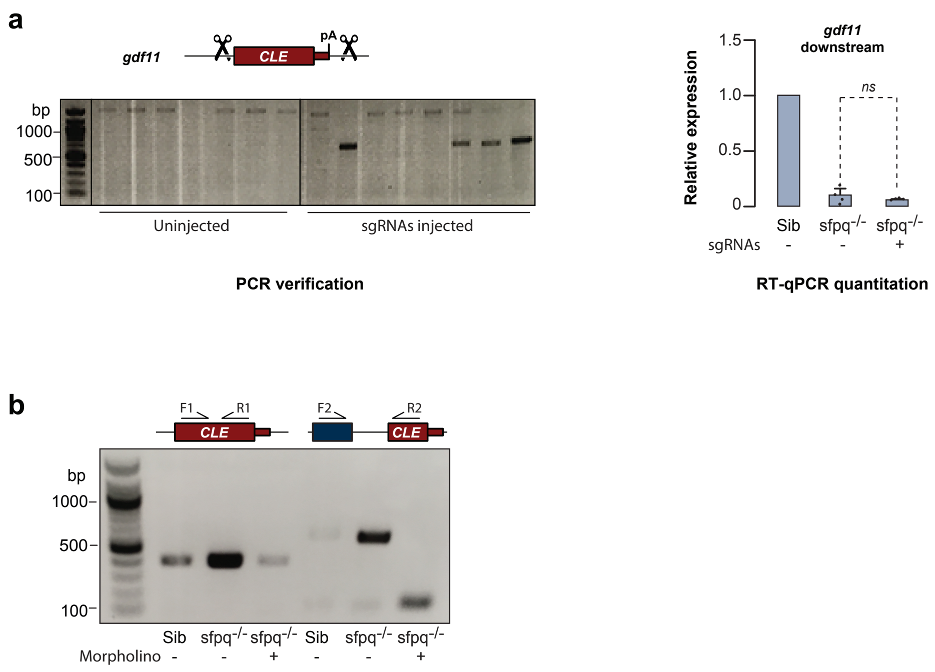

Supplementary Figure 5: Functional effects of CLEs in *sfpq-/-* embryos

a, Deletion of the *gdf11* CLE using CRISPR/Cas9 does not affect expression levels of the longer isoform. Left: PCR verification of Cas9 cleavage after injection of the *gdf11* sgRNAs. Representative image; experiment performed 4 times. Right: RT-qPCR quantitation of the relative expression of the downstream *gdf11* exons in *sfpq-/-* embryos compared to siblings (± SD); n= 4 biologically independent replicates

b, The *epha4b* splice-junction morpholino prevents expression of the cryptic exon in *sfpq-/-* embryos. Representative image; experiment performed 3 times.

Source data are provided as Source Data file.

S

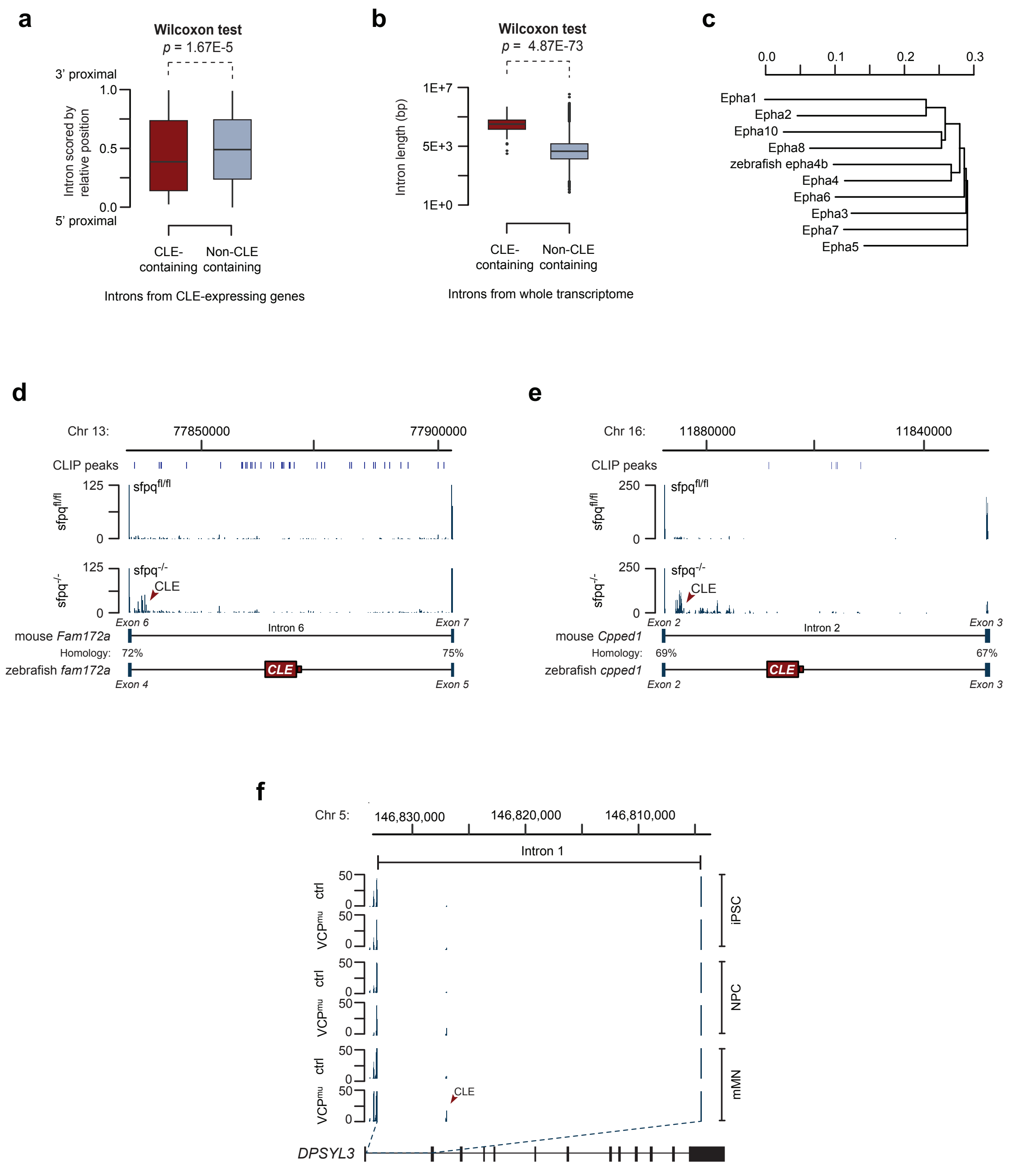

(Supplementary Figure 6. See next page for legend)

Supplementary Figure 6: The CLE-repressing function of SFPQ is conserved in mouse and in human neuropathology model

a, CLE containing (n = 144) and non-CLE containing introns (n = 2217) from mouse CLE-expressing genes were scored by its relative position in its gene and the distribution of these scores were plotted. The box bounds represent the first and third quartiles and the black lines at the middle of the boxes show the medians. Top and/or bottom whiskers represent 1.5x of the range between the third and the first quartiles (interquartile range). Two-sided Wilcoxon rank sum test was performed.

b, Distribution of the intron sizes from CLE-containing (n = 144) and non-CLE-containing introns (n = 2217). The box bounds represent the first and third quartiles and the black lines at the middle of the boxes show the medians. Top and/or bottom whiskers represent 1.5x of the range between the third and the first quartiles (interquartile range). Circles represent outliers. Two-sided Wilcoxon rank sum test was performed.

c, Phylogenetic relationship between mRNA sequence of zebrafish *epha4b* and paralogues of mouse *Epha* gene.

d, CLIP-seq peak distribution (top) and RNA-seq coverage plots (mid) from representative CLE-containing introns. Range of read coverage displayed is adjusted to magnify CLE/intronic reads. Sequence homology of flanking exons from orthologous CLE-containing genes are compared and the relative position of zebrafish CLE is shown (bottom)

e, Representative RNA-seq coverage plots from ALS-derived iPSC dataset of CLEs up-regulated in VCP^{mu} samples.
