# Supplementary material for: A conserved role for the ALS-linked splicing factor SFPQ in repression of pathogenic cryptic last exons
Source: Nat Commun. 2021 Mar 26;12:1918. doi: 10.1038/s41467-021-22098-z (PMC7997972; doi:10.1038/s41467-021-22098-z)
Supplement: Supplementary file 12 — Description of Additional Supplementary Files [file 41467_2021_22098_MOESM12_ESM.docx]

Description of additional supplementary information

Title: Supplementary Data 1

Description: List of GO terms in upregulated genes. Exact p-value was derived using minimum hypergeometric (mHG) statistical framework.

Title: Supplementary Data 2

Description: List of GO terms in down-regulated genes. Exact p-value was derived using minimum hypergeometric (mHG) statistical framework.

Title: Supplementary Data 3

Description: List of alternatively spliced genes from Cufflinks. Column qvalue represents FDR-adjusted p-value derived from Jensen-Shannon divergence

Title: Supplementary Data 4

Description: List of GO terms in splice variants. Exact p-value was derived using minimum hypergeometric (mHG) statistical framework.

Title: Supplementary Data 5

Description: List of CLEs from whippet

Title: Supplementary Data 6

Description: Functional consequence of CLE isoforms

Title: Supplementary Data 7

Description: List of CLEs from mouse Sfpq cKO model

Title: Supplementary Data 8

Description: List of CLEs from ALS-derived iPSCs

Title: Supplementary Data 9

Description: DNA oligonucleotides used in this study
