# Supplementary material for: A conserved role for the ALS-linked splicing factor SFPQ in repression of pathogenic cryptic last exons
Source: Nat Commun. 2021 Mar 26;12:1918. doi: 10.1038/s41467-021-22098-z (PMC7997972; doi:10.1038/s41467-021-22098-z)
Supplement: Supplementary file 13 — Reporting Summary [file 41467_2021_22098_MOESM13_ESM.pdf]

Corresponding author(s): Patricia Gordon, Corinne Houart

Last updated by author(s): Jan 25, 2021

Reporting Summary

Nature Research wishes to improve the reproducibility of the work that we publish. This form provides structure for consistency and transparency in reporting. For further information on Nature Research policies, see our Editorial Policies and the Editorial Policy Checklist.

Statistics

For all statistical analyses, confirm that the following items are present in the figure legend, table legend, main text, or Methods section.

n/a	Confirmed	
☐	☒	The exact sample size (*n*) for each experimental group/condition, given as a discrete number and unit of measurement
☐	☒	A statement on whether measurements were taken from distinct samples or whether the same sample was measured repeatedly
☐	☒	The statistical test(s) used AND whether they are one- or two-sided<br>*Only common tests should be described solely by name; describe more complex techniques in the Methods section.*
☐	☒	A description of all covariates tested
☐	☒	A description of any assumptions or corrections, such as tests of normality and adjustment for multiple comparisons
☐	☒	A full description of the statistical parameters including central tendency (e.g. means) or other basic estimates (e.g. regression coefficient) AND variation (e.g. standard deviation) or associated estimates of uncertainty (e.g. confidence intervals)
☐	☒	For null hypothesis testing, the test statistic (e.g. *F*, *t*, *r*) with confidence intervals, effect sizes, degrees of freedom and *P* value noted<br>*Give P values as exact values whenever suitable.*
☒	☐	For Bayesian analysis, information on the choice of priors and Markov chain Monte Carlo settings
☒	☐	For hierarchical and complex designs, identification of the appropriate level for tests and full reporting of outcomes
☐	☒	Estimates of effect sizes (e.g. Cohen's *d*, Pearson's *r*), indicating how they were calculated

Our web collection on statistics for biologists contains articles on many of the points above.

Software and code

Policy information about availability of computer code

Data collection

The main program we used for collecting data was:
- LightCycler 96 software [Version 1.1.0.1320]; Roche

Data analysis

Computer code used in this study is described in the Methods

The main software that we used were:
- Bowtie2 (Version 2.2.6); (Langmead and Salzberg, 2012); http://bowtie-bio.sourceforge.net/bowtie2/index.shtml
- Cufflinks (Version 2.2.1); (Trapnell et al, 2012); http://cole-trapnell-lab.github.io/cufflinks/manual/
- Whippet (Version 0.11.1); (Timothy Sterne-Weiler et al, 2018); https://github.com/timbitz/Whippet.jl
- BBDuk (Version 38.87); (Joint Genome Institute); https://jgi.doe.gov/data-and-tools/bbtools/bb-tools-user-guide/bbduk-guide/
- HISAT2 (Version 2.1.0); (Kim et al, 2015); http://ccb.jhu.edu/software/hisat2/index.shtml
- StringTie2 (Version 2.1.3); (Kovaka et al, 2019); https://ccb.jhu.edu/software/stringtie/
- GenomicRanges (Version 3.12); (Lawrence et al, 2013); https://bioconductor.org/packages/release/bioc/html/GenomicRanges.html
- GenomicFeatures (Version 3.12); (Lawrence et al, 2013); https://bioconductor.org/packages/release/bioc/html/GenomicFeatures.html
- MEME Suite (Version 5.3.0); (Timothy et al, 2009); http://meme-suite.org/
- MaxEntScan (Version 0_2004.04.21-2); (Yeo and Burge, 2003);http://hollywood.mit.edu/burgelab/maxent/Xmaxentscan_scoreseq.html
- WebLogo(Version 2.8.2); (Crooks et al, 2004); https://weblogo.berkeley.edu/logo.cgi
- R (Version 4.0.3); (RCoreTeam, 2020); https://www.r-project.org/
- edgeR (Version 3.12.1); (Robinson et al 2010); https://www.bioconductor.org/packages/release/bioc/html/edgeR.html
- IGV (Version 2.4); (Robinson et al, 2011); https://software.broadinstitute.org/software/igv/download

For manuscripts utilizing custom algorithms or software that are central to the research but not yet described in published literature, software must be made available to editors and reviewers. We strongly encourage code deposition in a community repository (e.g. GitHub). See the Nature Research guidelines for submitting code & software for further information.

Data

Policy information about availability of data

All manuscripts must include a data availability statement. This statement should provide the following information, where applicable:

- Accession codes, unique identifiers, or web links for publicly available datasets
- A list of figures that have associated raw data
- A description of any restrictions on data availability

The RNA-seq data analyzed in Figures 1 and 3 is available at ArrayExpress, accession number E-MTAB-9113 (https://www.ebi.ac.uk/arrayexpress/experiments/E-MTAB-9113/)
The 3' mRNA-seq data analyzed in Figure 2 is available at ArrayExpress, accession number E-MTAB-9899 (https://www.ebi.ac.uk/arrayexpress/experiments/E-MTAB-9899/).

Field-specific reporting

Please select the one below that is the best fit for your research. If you are not sure, read the appropriate sections before making your selection.

☒ Life sciences ☐ Behavioural & social sciences ☐ Ecological, evolutionary & environmental sciences

For a reference copy of the document with all sections, see nature.com/documents/nr-reporting-summary-flat.pdf

Life sciences study design

All studies must disclose on these points even when the disclosure is negative.

Sample size	No sample-size calculations were performed. All experiments were performed with at least n=3.
Data exclusions	No data was excluded
Replication	Experiments were reproduced at least once, as indicated in the manuscript. No experiments failed to reproduce.
Randomization	Sample allocation was random
Blinding	Investigators were not blinded during data collection and analysis. The morphological differences in sfpq-/- embryos make them visually distinct from wildtype embryos, thus investigators can easily determine which embryos they are looking at.

Reporting for specific materials, systems and methods

We require information from authors about some types of materials, experimental systems and methods used in many studies. Here, indicate whether each material, system or method listed is relevant to your study. If you are not sure if a list item applies to your research, read the appropriate section before selecting a response.

Materials & experimental systems

n/a	Involved in the study	
☐	☒	Antibodies
☒	☐	Eukaryotic cell lines
☒	☐	Palaeontology and archaeology
☐	☒	Animals and other organisms
☒	☐	Human research participants
☒	☐	Clinical data
☒	☐	Dual use research of concern

Methods

n/a	Involved in the study	
☒	☐	ChIP-seq
☒	☐	Flow cytometry
☒	☐	MRI-based neuroimaging

Antibodies

Antibodies used	anti-SFPQ: Abcam ab38148,<br>anti-IgG: Thermo-Fisher MA5-14453
Validation	The sfpq antibody was validated by Western blot in wildtype and mutant embryos. See also our previous work with this antibody: Thomas-Jinu et al, "Non-nuclear Pool of Splicing Factor SFPQ Regulates Axonal Transcripts Required for Normal Motor Development", Neuron 2017.

Animals and other organisms

Policy information about studies involving animals; ARRIVE guidelines recommended for reporting animal research

Laboratory animals	Zebrafish of the AB wildtype and sfpq-kg41 strains were used. Adults were only used for breeding, and embryos were analyzed before 30 hpf
Wild animals	No wild animals were used
Field-collected samples	No samples were collected from the field
Ethics oversight	Zebrafish procedures were approved by the UK Home Office in accordance with the Animals (Scientific Procedures) Act 1986, under CH Home Office Project license 70/7577

Note that full information on the approval of the study protocol must also be provided in the manuscript.
